# Geo-epidemiology of animal tuberculosis and *Mycobacterium bovis* genotypes in livestock in a small, high-incidence area in Sicily, Italy

**DOI:** 10.3389/fmicb.2023.1107396

**Published:** 2023-03-17

**Authors:** Cinzia Marianelli, Vladimiro Verrubbi, Flavia Pruiti Ciarello, Dorotea Ippolito, Maria Lodovica Pacciarini, Vincenzo Di Marco Lo Presti

**Affiliations:** ^1^Department of Food Safety, Nutrition and Veterinary Public Health, Istituto Superiore di Sanità, Rome, Italy; ^2^Agenzia nazionale per le nuove tecnologie, l’energia e lo sviluppo economico sostenibile, Rome, Italy; ^3^Istituto Zooprofilattico Sperimentale della Sicilia, Sezione Diagnostica Barcellona Pozzo di Gotto, Barcellona Pozzo di Gotto, Italy; ^4^National Reference Centre for Bovine Tuberculosis, Istituto Zooprofilattico Sperimentale della Lombardia e dell’Emilia Romagna, Brescia, Italy

**Keywords:** geo-epidemiology, *Mycobacterium bovis*, genetic analysis, landscape structure, livestock

## Abstract

**Introduction:**

The persistence of animal tuberculosis (TB) in livestock is a major concern in Sicily, Italy. The objective of this study was to elucidate the transmission dynamics of *M. bovis* infection in a highly circumscribed, and at the same time geographically diverse, high-risk area of the island through an in-depth geo-epidemiological investigation of TB in cattle and black pigs raised in small-scale extensive farms across the district of Caronia.

**Methods:**

We used genotype analysis coupled with geographic information system (GIS) technology and phylogenetic inference to characterize the spatial distribution of TB and *M. bovis* genotypes in livestock and the genetic relationships between *M. bovis* isolates. A total of 589 *M. bovis* isolates collected from slaughtered cattle (*n* = 527) and Sicilian black pigs (*n* = 62) over a 5-year period (2014–2018) were included in the study.

**Results:**

TB was widespread throughout the district and was most frequent in the north-central area of the district, especially along one of the district’s streams. We identified a total of 62 *M. bovis* genotypes. Identical genetic profiles were isolated from both neighboring and non-neighburing herds. The 10 most frequent genotypes, accounting for 82% of *M. bovis* isolates, showed geographic specificities in that they tended to cluster in specific spatial niches. The landscape structure of these niches—i.e. steep slopes, rocky ridges, meadows and streams—is likely to have had a significant influence on the distribution of TB among livestock in Caronia. Higher concentrations of TB were observed along streams and in open meadows, while rocky ridges and slopes appeared to have hampered the spread of TB.

**Discussion:**

The geographical distribution of TB cases among livestock in Caronia is consistent with several epidemiological scenarios (e.g., high density of infected herds along the streams or in hilly plateau where livestock share pastures). Landscape structure is likely to play an important role in the transmission and persistence of *M. bovis* infection across the district. Additional potential risk factors, such as livestock trading and extensive breeding methods, are also discussed. Our results will contribute to the improvement of surveillance, control and eradication activities of TB in Sicily by the implementation of *ad hoc* TB control measures, especially in farms located along streams, sharing common pastures or with mixed animal species.

## 1. Introduction

Animal tuberculosis (TB) is an infectious disease mainly caused by *Mycobacterium bovis*, which has a wide host range affecting both domestic and wild animals, as well as humans. Cattle is the main host species for *M. bovis*. However, several wildlife hosts—i.e. badger, red deer, wild boar, brushtail possum—can carry and spread the pathogen to livestock (Palmer et al., 2012; Fitzgerald and Kaneene, 2013).

Infected animals with progressive disease shed the bacteria in mucus, saliva, aerosols, feces, milk, and sometimes in urine, vaginal secretions, or semen. TB can thus be transmitted by a number of routes, most commonly through inhalation, through contact with the excreta of infected animals (Phillips et al., 2003), as well as the ingestion of contaminated food and water (Allen et al., 2021). *Mycobacterium bovis* can survive in water and soil for long periods (Fine et al., 2011), raising the risk of infection.

*Mycobacterium bovis* represents a serious public health concern in both industrialized and non-industrialized countries, where the absence or inadequacy of TB control programs, immunodeficiency, malnutrition, or the adoption of social and cultural behaviors (e.g., close contact with animals and consumption of animal products, improper cooking practices and poor hygiene) increase the risk of human infection (Malama et al., 2013; Olea-Popelka et al., 2017; Mohamed, 2019). *Mycobacterium bovis* is also one of the biggest challenges facing rural communities and the farming industry, as it causes both direct and indirect economic losses. It increases morbidity and mortality, lowers milk and meat production, reduces fertility and hampers the commercial trade in animals (Pérez-Morote et al., 2020).

Molecular epidemiology based on genetic profiles of *M. bovis*, which consists of spoligotypes and mycobacterial interspersed repetitive unit-variable number tandem repeat codes (MIRU-VNTR), is recognized as a valuable tool for the identification of sources of infection, routes of transmission and host preference, and the understanding of *M. bovis* transmission dynamics within a population or an ecosystem (El-Sayed et al., 2016). In recent years, web technologies and geo-epidemiology based on Geographic Information System (GIS) technology, have emerged as useful tools in epidemiology to monitor several diseases in time and space, including human (Beiranvand et al., 2016; Gehlen et al., 2019) and animal (Zaragoza Bastida et al., 2012; Hauer et al., 2015; Proud Tembo et al., 2020; Reis et al., 2020; Skuce et al., 2020; Almaw et al., 2021; Reis et al., 2021) TB.

In Sicily, the largest island in the Mediterranean and one of Italy’s 20 regions, TB is still a major concern despite the implementation of state and local eradication programs for TB in cattle. Our group has recently conducted two molecular-epidemiological investigations based on the genotyping of *M. bovis* isolates from livestock and wild animals, first throughout the island (Amato et al., 2018), and subsequently in the province of Messina, in northeastern Sicily (Marianelli et al., 2019). A large variety of *M. bovis* strains, which were in several cases common to different species of livestock, were identified (Amato et al., 2018; Marianelli et al., 2019). Livestock reared in the district of Caronia, situated in the province of Messina, showed the highest share of TB both in the island as a whole, and in the province (Amato et al., 2018; Marianelli et al., 2019). More than 50% of TB cases in the province of Messina were isolated from Caronia-bred livestock (Marianelli et al., 2019).

To shed light on the potential risk factors affecting the persistence of *M. bovis* infection in livestock in the district of Caronia – we conducted an in-depth geo-epidemiological investigation, combining genotyping, geospatial (GIS), and phylogenetic analyses to investigate the distribution of TB and *M. bovis* genotypes in livestock, the genetic relationships among *M. bovis* isolates, the transmission dynamics of *M. bovis* infection and the role played by the ecosystem in the spread of the pathogen in livestock in this highly circumscribed, and at the same time geographically diverse, high-incidence area.

## 2. Materials and methods

### 2.1. Area under study

**Figure 1:**
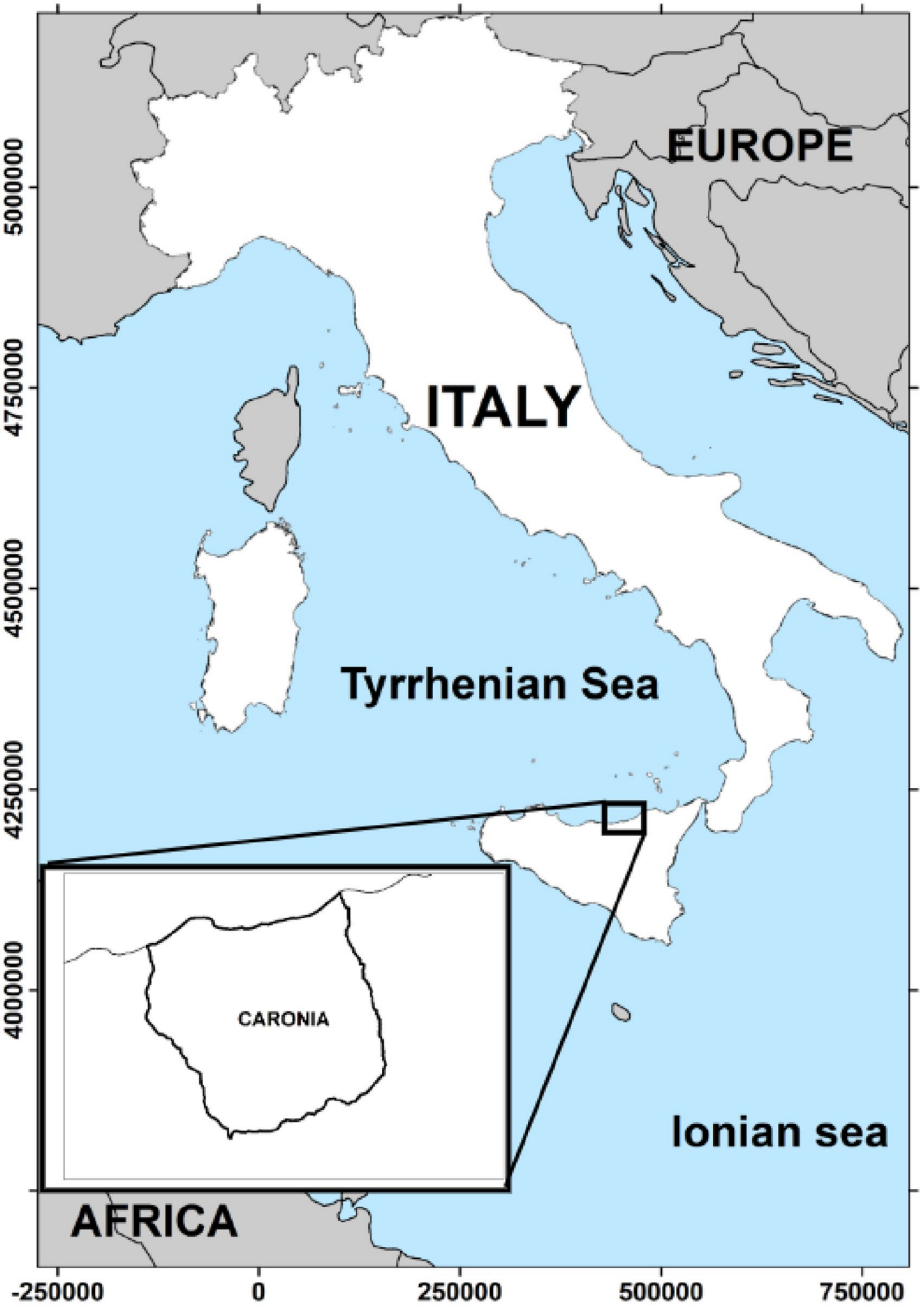
The geographical area under study. The district of Caronia covers 227.3 km^2^ in the province of Messina on the northern coast of Sicily, Italy. The map was adapted from QGis.

The district of Caronia is a very small area, 227.3 km^2^, in the province of Messina on the northern coast of Sicily, Italy (Figure 1). The area is part of the Nebrodi Park, a rural nature reserve, and consists mainly of mountainous terrain with several peaks exceeding 1,500 m in altitude, the Nebrodi mountains. The north-facing slopes, covered in olive and citrus groves, descend gently toward the Tyrrhenian Sea. Caronia’s coast stretches for 20 km, lined with Mediterranean scrub. The catchment area consists of small streams—the Caronia, the Buzza, the Sampieri, and the San Barbaro Fughetto.

The district is heavily wooded. Cork oak, Turkey oak, holm oak and beech are the most abundant species. The woods give shelter to numerous species of birds, reptiles, amphibians and wild mammals, such as hedgehog, wildcat, marten and several small rodents (e.g., common dormouse, garden dormouse, European fat dormouse). Wild boar and fallow deer, which may act as natural reservoirs of TB (Gortazar et al., 2005; Naranjo et al., 2008; Martínez-López et al., 2014), are absent from the Nebrodi Park. Domestic farm animals like cattle, sheep, goats and Sicilian black pigs are reared in the area in numerous small-scale farms, with an average herd size of 40 animals per farm. Cattle, the most common and widespread kind of livestock in the area, is invariably raised under an extensive farming system, a system based on year-round grazing on natural pastures. The Sicilian black pig is an autochthonous breed of domestic pig, mostly reared in free or semi-free roaming conditions in the Park’s woods and natural pastures, throughout the year. These pastures are frequently shared with grazing cattle, as previously described (Di Marco et al., 2012).

### 2.2. Selection of TB cases in livestock

A total of 589 *M. bovis* isolates from slaughtered cattle (*n* = 527) and Sicilian black pigs (*n* = 62) were included in the study. All isolates were collected in the district of Caronia from 2014 to 2018. No animals were slaughtered for the purposes of this study and no permission was required for either slaughtering or sample collection.

The average annual size of livestock populations during the study period was of about 4,700 cattle and 4,600 black pigs.

Data collection for cattle was performed as part of a national surveillance required by law. In light of the high prevalence rate of TB in livestock in the area in 2013 (about 6.40%) and the high density of farms, a special 5-year surveillance program (2014–2018), was implemented for the control of TB in cattle. Throughout this period, skin and interferon-gamma testing of cattle were performed in addition to the routine slaughterhouse surveillance normally performed on all slaughtered livestock.

Identical protocols were implemented for black pigs: abattoir inspections as required by law, and *in-vivo* testing in the framework a series of research projects funded by the Italian Ministry of Health and the Ministry of Public Education.

Tissue samples from animals with a positive skin test on farms, and from animals with TB-compatible lesions upon routine abattoir inspections, were collected and cultured for mycobacterium at the diagnostic laboratory of the Istituto Zooprofilattico della Sicilia, Area Barcellona P.G., Messina. The individual animal was considered a TB case if the culture confirmed *M. bovis* infection. All *M. bovis* isolates from both cattle and black pigs were genotyped using spoligotyping and 12-locus MIRU-VNTR typing, as previously described (Marianelli et al., 2019). For MIRU-VNTR typing, 12 genomic loci were selected, according to Boniotti et al. (2009), and amplified individually: VNTR loci 2,165, 2,461, 0577, 0580 and 3,192 (i.e., ETR-A to –E; Frothingham and Meeker-O'Connell, 1998), VNTR locus 2,996 (i.e., MIRU26; Supply et al., 2000), VNTR loci 2163a, 2163b, 3,155 and 4,052 (Skuce et al., 2002), and VNTR loci 1,895 and 3,232 (Roring et al., 2002). *Mycobacterium tuberculosis* H37Rv was used as reference strain. Allele assignment was performed on the basis of PCR fragment size as compared to a 50-bp molecular weight marker. Results were stored in the TB database of the National Reference Centre for TB at Istituto Zooprofilattico Sperimentale della Lombardia e dell’Emilia Romagna, Brescia, Italy.

We extracted the above *M. bovis* genotype data—spoligotypes and 12-loci MIRU-VNTR codes—from the database, and combined them with each other to enhance the discriminatory power of the subsequent molecular epidemiological analysis. We also gathered and analyzed additional data, such as the type of host (cattle or Sicilian black pig), herd location (latitude and longitude coordinates reported by the farmers to the local authorities by law) and year of *M. bovis* isolation.

### 2.3. Spatial analysis

The QGis software, a free and open source GIS available at https://www.qgis.org/en/site/ (QGis Development Team 2020 version 3.18), was used to map farms and TB infections in the herds, as well as to study the geographic characteristics of the area under study and their possible relationship with TB occurrence data and *M. bovis* genotype frequencies in livestock.

### 2.4. Minimum spanning tree phylogenetic analysis

Twelve-locus MIRU-VNTR codes were used to infer relationships between isolates sharing the same spoligotypes. PHYLOViZ 2.0 (Nascimento et al., 2017-P1), a free online tool based on the goeBURST algorithm, was used to perform data analysis. The tool is available at https://online.phyloviz.net/index.

## 3. Results

### 3.1. Cases of TB in livestock

A total of 589 TB cases in livestock (*n* = 527 from cattle and *n* = 62 from black pigs) were included in the study.

*Mycobacterium bovis* infection involved 107 out of 236 small-scale farms (107/236, 45%) scattered across the district of Caronia. The majority of affected farms (73%) had multiple and recurrent infections over the study period. In many of them (42%), between 2 and 6 cases of TB were recorded (Table 1). The most highly infected herd had 28 cases of infection in cattle (the average annual size of the herd during the study period was 74 head of cattle).

**Table 1:** Number of infected farms and of *Mycobacterium bovis* isolates by number of cases per farm.

Number of TB cases per farm	Number of infected farms (*N* = 107)	*M. bovis* isolates (*N* = 589)
1	29	29
2–6	45	159
7–11	17	145
12–16	11	147
>17	5	109

### 3.2. Spatial distribution of TB-affected herds

**Figure 2:**
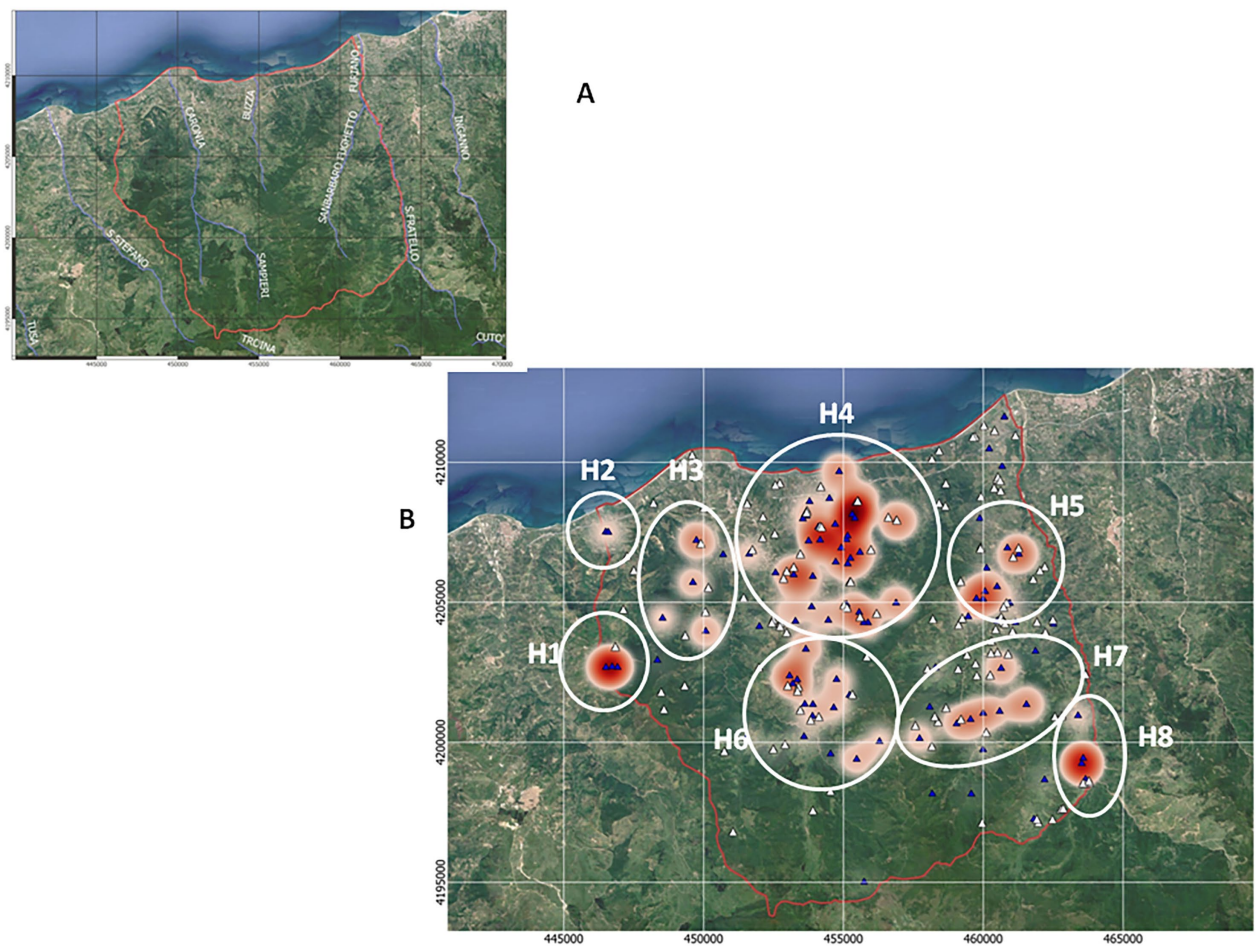
TB infections across the Caronia district. **(A)** The main streams flowing through the district—Caronia, Buzza, Sampieri, and San Barbaro. **(B)** Farms, both infected (*n* = 107) and uninfected (*n* = 129), are shown as blue and white triangles, respectively. Shades of red represent TB infections, where darker red represents a higher number of cases per herd. The eight geographical subareas of TB infection (H1–H8) are circled. The boundaries of the district of Caronia is indicated (the red line). Both maps were adapted from QGis.

The main streams and all of Caronia’s farms (*n* = 236), both infected (*n* = 107) and uninfected (*n* = 129), were localized using the QGis software (Figures 2A,B). The number of TB infections per farm is also shown, as shades of red (Figure 2B).

**Figure 3:**
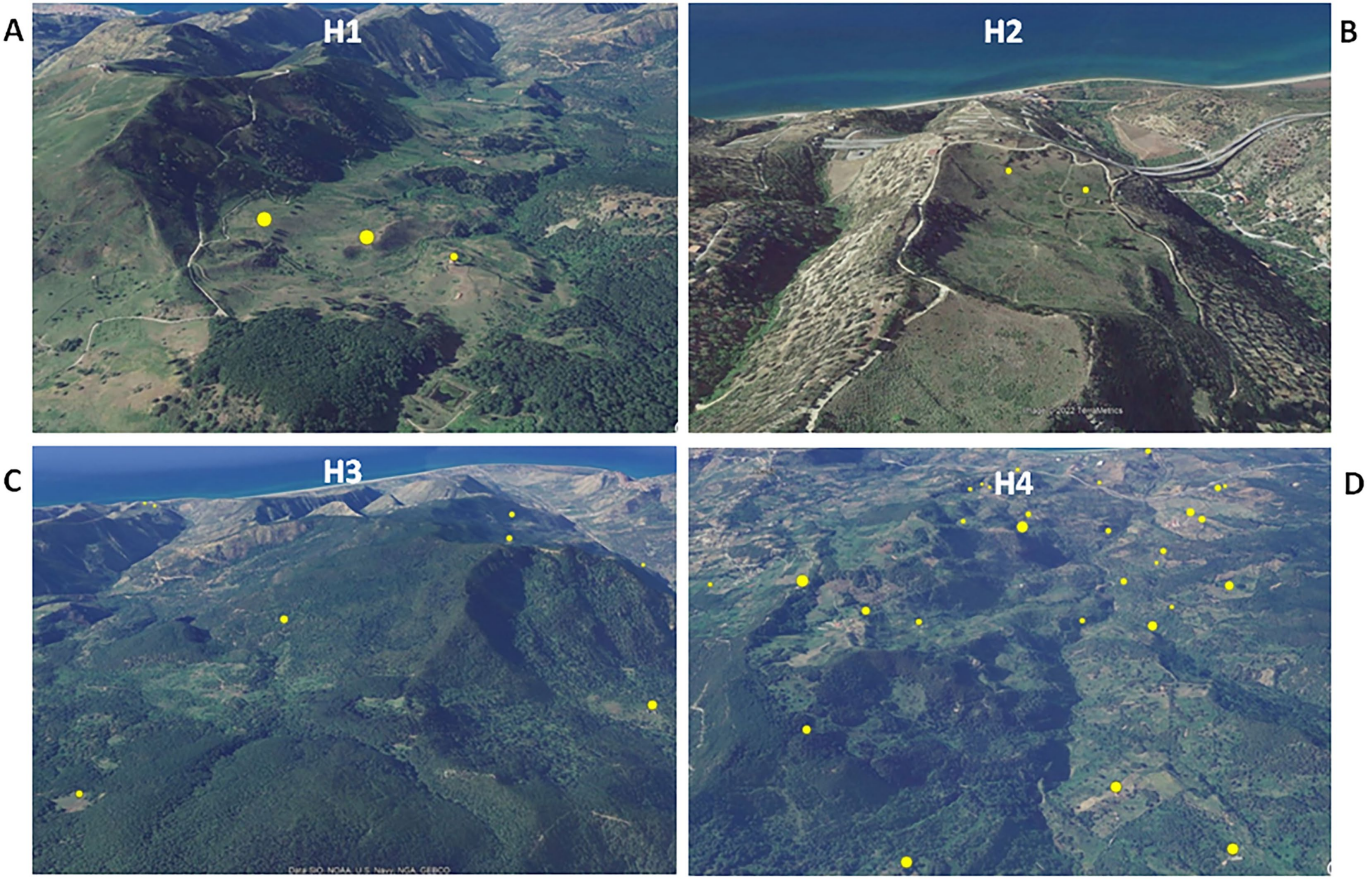
Satellite pictures of subareas H1–H4 **(A–D)**. Infected herds are shown as yellow dots the size of which is proportional to the number of infected animals per herd. The maps were adapted from QGis.

**Figure 4:**
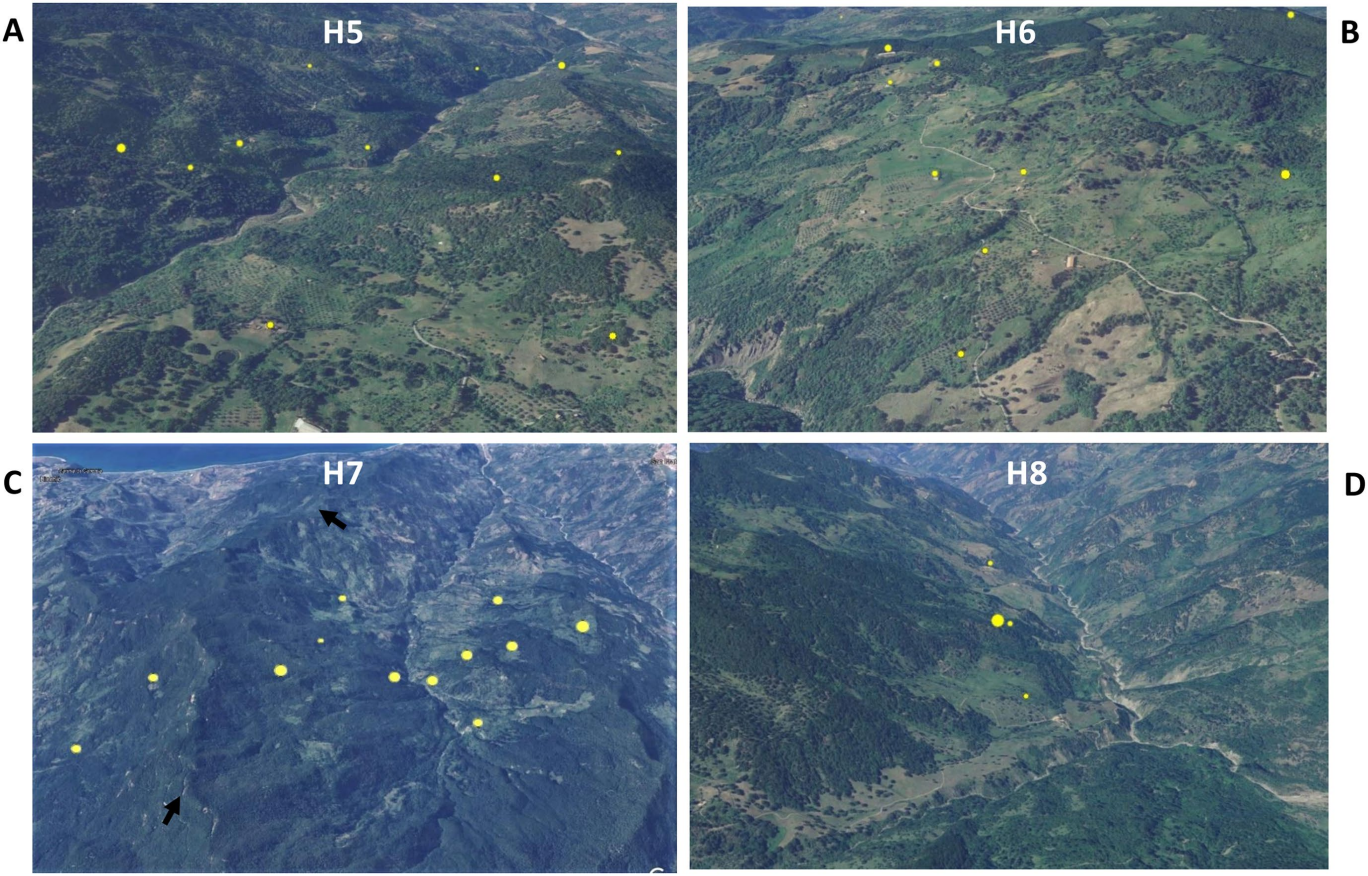
Satellite pictures of subareas H5–H8 **(A–D)**. Infected herds are shown as yellow dots the size of which is proportional to the number of infected animals per herd. The black arrows **(C)** indicate a rocky ridge. The maps were adapted from QGis.

Animal tuberculosis was widespread across Caronia. The disease was not randomly distributed across the district, however, but rather clustered in specific areas, mainly in the north-central area along the Buzza stream (Figure 2, red spots). Analyzing the landscape structure of Caronia, we identified eight major spatial niches or subareas—marked from H1 to H8—in which neighboring infected herds clustered. This identification was based on the density of cases in defined areas (Figure 2). The landscape morphology of subareas H1-H4 and H5-H8 is shown in Figures 3A–D, 4A–D.

Subarea H1 comprised three neighboring infected farms located on an intermontane plateau at about 700 m above sea level (a.s.l; Figure 3A). Subarea H2 comprised two neighboring infected farms on a meadow at about 200 m a.s.l, partially surrounded by slopes and streets (Figure 3B). Subarea H3 involved several infected herds across a large, heavily forested area west of the Caronia stream at 600–800 m a.s.l (Figure 3C). Subarea H4, which accounted for most TB cases, consisted of numerous infected herds along the Buzza stream across a large hilly terrain at 100–500 m a.s.l, where wooded and bushy areas alternate with farms and cultivated fields (Figure 3D). Subarea H5 comprised several infected herds along the San Barbaro Fughetto stream in a large area covered in bushes and trees at 200–600 a.s.l (Figure 4A). Subarea H6 comprised a few infected farms located in a stretch of land north-east of the Sampieri stream, with bushes and trees interspersed with cultivated fields, at 500–1,000 m a.s.l (Figure 4B). Subarea H7 encompassed about 10 infected herds situated in a hilly plateau, east of a rocky ridge, with bushes and trees at 600–900 m a.s.l (Figure 4C). Finally, subarea H8 comprised a small number of neighboring, infected herds located close to the San Fratello stream in a wooded area (Figure 4D). Subareas H5, H7, and H8, all situated on the eastern side of the district, border to the west on a long rocky ridge 800–1,000 m a.s.l, visible in Figure 4C.

### 3.3. Genotypes of *Mycobacterium bovis* isolates

Genotype data—spoligotypes and 12 loci-MIRU-VNTR codes—of all *M. bovis* (*n* = 589) isolated over the 5-year study, were combined to enhance the discriminatory power of the molecular epidemiological analysis. A total of 11 spoligotypes were found: SB0120, SB0133, SB0134, SB0841, SB0850, SB1167, SB1305, SB1564, SB1566, SB2368 and SB2473. Overall, SB0120, SB0134 and SB0841 were the most frequent, accounting for 49% (286/589), 21% (125/589), and 20% (116/589) of *M. bovis* isolates, respectively. MIRU-VNTR analysis yielded a total of 50 MIRU-VNTR codes. The combination of spoligotypes and MIRU-VNTR codes increased the number of genetic profiles to 62.

**Figure 5:**
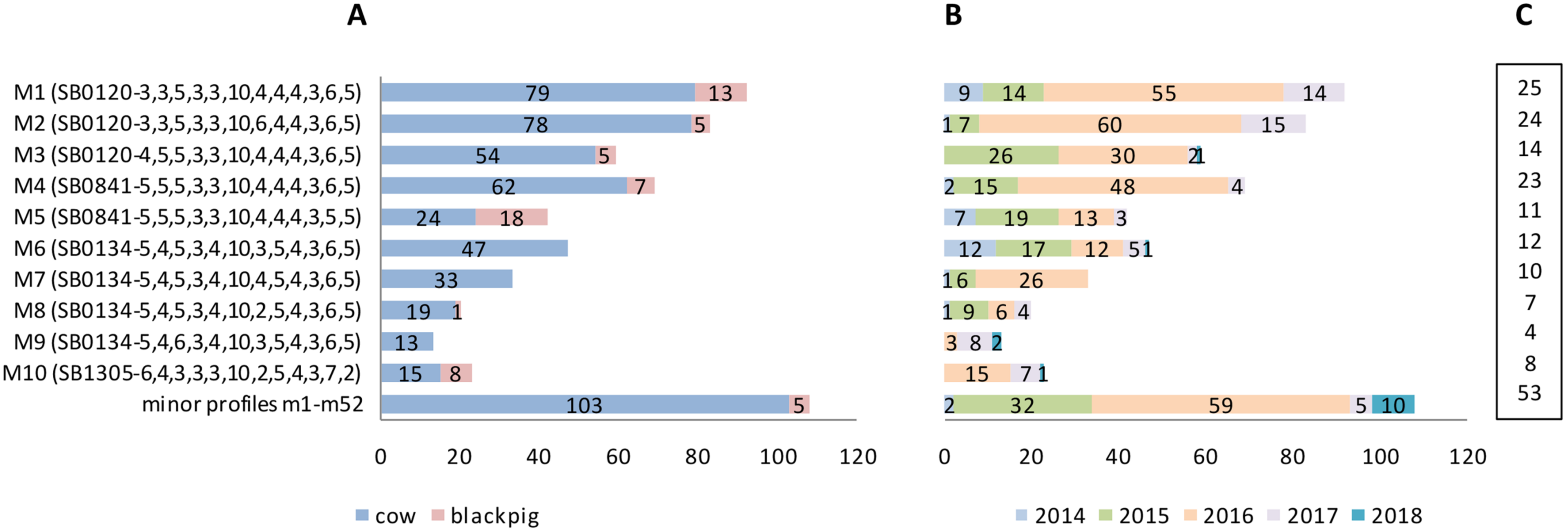
*Mycobacterium bovis* genetic profiles by host, year of isolation and number of infected farms. **(A)** The top 10 most frequently isolated profiles (major profiles M1–M10) and the group of less frequent, minor profiles (m1–m52, further details in Supplementary Table S1 and section 3.3.2) by host: number of isolates from cattle in blue, black pigs in pink. **(B)** Genetic profiles by year of isolation (number of isolates). **(C)** Genetic profiles by number of TB infected farms.

Of these 62 combined genetic profiles, 10 were the most frequent and accounted for 82% *M. bovis* isolates (481/589). These 10 profiles are hereinafter referred to as “the major profiles.” The remaining 52 genetic profiles, accounting for 18% *M. bovis* isolates (108/589), are hereinafter referred to as “the minor profiles.” Results are shown in Figure 5.

#### 3.3.1. Major genetic profiles

The 10 major genetic profiles, which accounted for 481 *M. bovis* isolates (481/589; 82%), were isolated both from cattle (*n* = 424) and from black pigs (*n* = 57). These profiles—labeled M1–M10—comprised spoligotypes SB0120 (three profiles), SB0841 (two profiles), SB0134 (four profiles), and SB1305 (one profile) with their respective MIRU-VNTR codes as detailed in Figure 5A. The remaining 52 minor profiles appear at the bottom of Figure 5A as a single group, and are described in section 3.3.2 below.

Most of the major profiles (7/10; 70%—M1–M5, M8, and M10) affected both kinds of livestock. However, with the exception of profile M5, which infected cattle and black pigs almost equally, all other profiles prevailed in cattle. The three major profiles M6, M7, and M9 affected only cattle (Figure 5A).

The highest number of TB cases was recorded in 2015 and 2016, with a total of 145 and 327 *M. bovis* isolates, respectively (Figure 5B). M1, M2, and M4 were the most frequent major profiles and affected more than 20 farms each (Figure 5C).

#### 3.3.2. Minor genetic profiles

The 52 minor genetic profiles, accounting for 18% (108/589) of *M. bovis* isolates, were isolated almost exclusively from cattle (cattle *n* = 103; black pigs *n* = 5). These profiles—named m1–m52—are detailed in Supplementary Table S1. Here, too, as with the major profiles, SB0120 was the most frequent spoligotype, which generated, in combination with various MIRU-VNTR codes, 31 different minor profiles.

Most of the minor profiles (41/52; 79%) affected only one or two individual animals (Supplementary Table S1). Profiles m49 and m50, however, caused the highest number of cases, affecting a total of 11 and 10 cattle, respectively. These same two profiles also involved the highest number of farms—eight and four herds, respectively. As with the major profiles, nearly all minor profiles were isolated in 2015 and 2016 (Supplementary Table S1).

### 3.4. Geographical distribution of *Mycobacterium bovis* genotypes

Major profiles M1–M10 were identified in 90 of 107 infected farms (84%), and minor profiles were isolated from 53 of 107 infected farms (50%). Specifically, 54 herds were affected only by major profiles, 17 herds were affected only by minor profiles and 36 herds were affected by both major and minor profiles.

**Figure 6:**
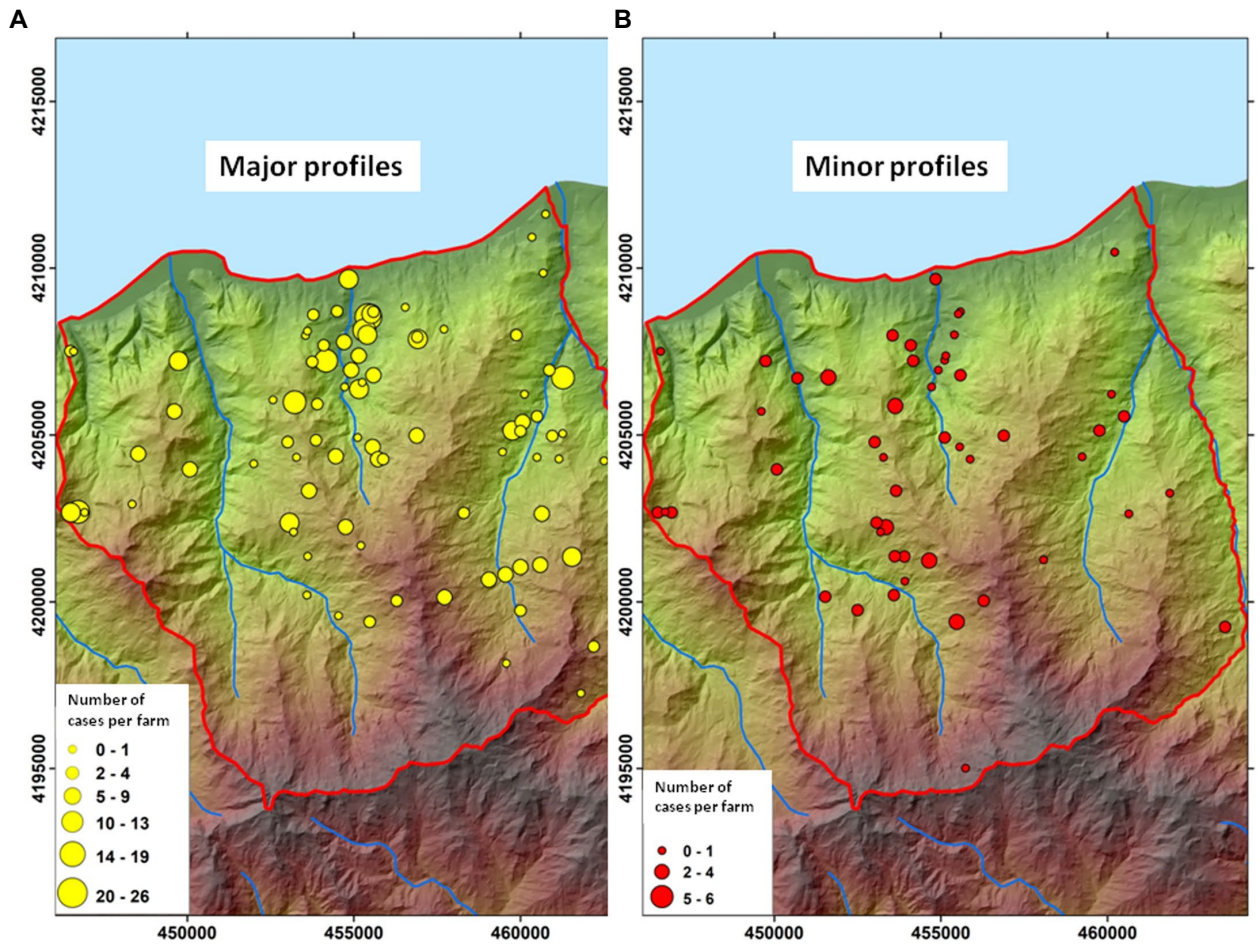
Geographic distribution of *Mycobacterium bovis* genotypes. **(A)** farms affected by major *M. bovis* profiles (*n* = 90). **(B)** Farms affected by minor *M. bovis* profiles (*n* = 53). The size of the dots is proportional to the number of infected animals (cattle and black pigs) per herd. Both maps were adapted from QGis.

The herds affected by major (*n* = 90) and minor profiles (*n* = 53) are mapped in Figures 6A,B, respectively. In both cases, profiles grouped mostly in the north-central area of the Caronia district along the Buzza stream.

**Figure 7:**
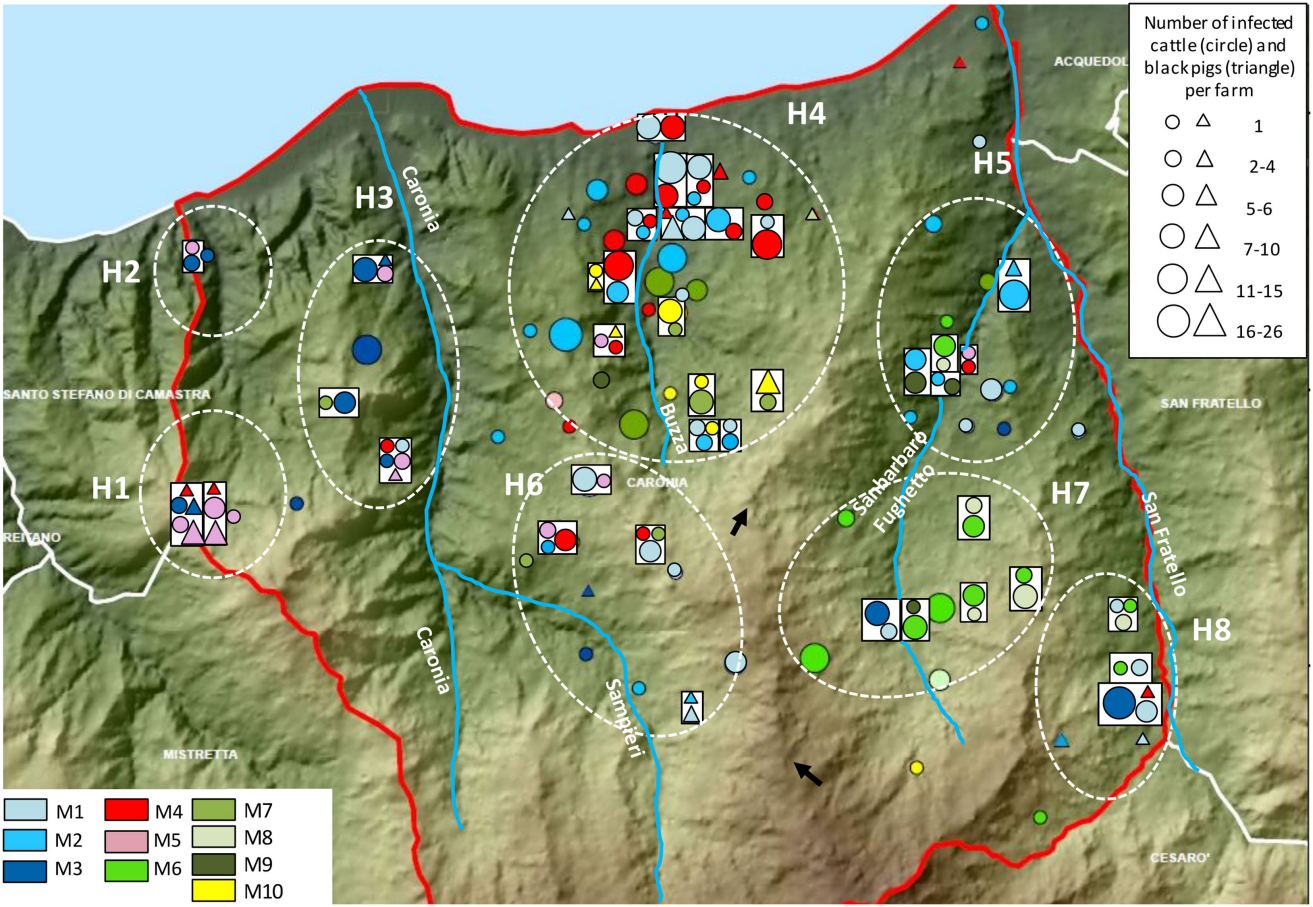
Distribution and frequency of the major genetic profiles of *Mycobacterium bovis*. Cattle and black pigs are marked as circles and triangles, respectively. The size of the circle and triangle symbols is proportional to the number of infected animals per herd. Symbols inside rectangles refer to the same herd. The black arrows indicate a rocky ridge. The Caronia, Sampieri, Buzza and Sanbarbaro Fughetto streams are indicated, as well as subareas H1–H8. The map was adapted from QGis.

The distribution and the frequency of the major profiles are shown in Figure 7. Numerous genetic profiles were common to cattle and/or black pigs from both neighboring and distant herds. Major profiles appeared to cluster in distinct subareas. M3 and M5 clustered mostly in the western part of the district in subareas H1, H2, and H3. Profiles M1, M2, and M4 emerged largely in the north-central area, in subarea H4, and profiles M7 and M10 just south of them, in the same subarea, with M10 mapping almost entirely in this subarea. M6, M8, and M9 appeared exclusively in the south-eastern territory, east of a rocky ridge, and predominantly grouped within subarea H7.

Not surprisingly, identical genetic profiles were found mostly in livestock originating from the same or neighboring herds. However, as shown in Figure 7, some non-neighboring herds exhibited identical profiles as well. Neighboring and non-neighboring herds from H1-H3 shared profiles M3 and M5 almost exclusively. Herds from H7 were predominantly affected by profiles M6 and M8, both of which were also present in subareas H5 and H8. Subareas H5, H7, and H8 are all located in the eastern part of the district, east of a rocky ridge as indicated by the black arrows in Figure 7. Subareas H4–H6 and H8 displayed many different genetic profiles: M1, M2, M4, M7, and M10 were recorded mainly in H4, M1 prevailed in H6 and H8, and M2 predominated in H5.

As noted, non-neighboring herds shared common profiles. M1 was recorded mainly in H4 but appeared also in H3, H5, H6, and H8. M2 and M4 were detected for the most part in H4; however, a few cases were also found in H5 and H6. M4 was identified in H1 and H3 as well. M3 and M5 prevailed in the western part of the Caronia district, but a few cases of M3 were documented also in the southern (H6) and eastern (H5, H7, and H8) areas, and M5 was also found in H4, H5 and H6. M6 and M8 were predominant in the western part of Caronia and were isolated from livestock in H5, H7, and H8. M7 was recorded mainly in H4 but also in H5 and H6. M9 was detected in H4, H5, and H7. Finally, M10 was detected almost exclusively in H4. One case, however, was recorded in the south of the province (Figure 7).

**Figure 8:**
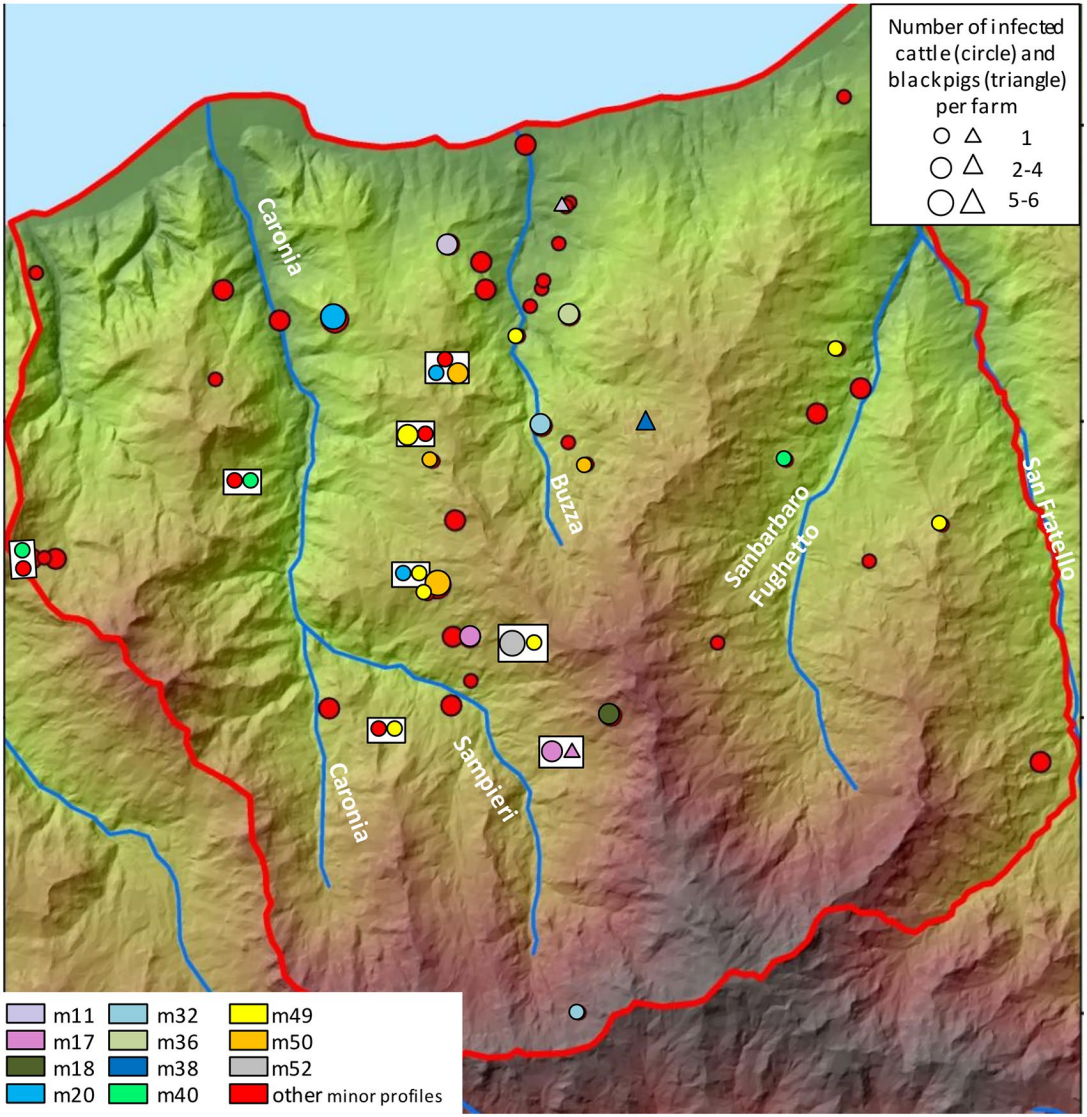
Distribution and frequency of the minor genetic profiles of *Mycobacterium bovis*. Cattle and black pigs are marked as circles and triangles, respectively. The size of the circle and triangle symbols is proportional to the number of infected animals per herd. Symbols inside rectangles refer to the same herd. The Caronia, Sampieri, Buzza and Sanbarbaro Fughetto streams are indicated. The map was adapted from QGis.

The distribution and frequency of minor profiles are shown in Figure 8, although only profiles affecting more than two animals have been assigned distinct colors. While minor profiles did not seem to cluster in specific spatial niches, both m49 and m50—the most frequent—mapped in the central area. Profile m49 was also recorded in the eastern part of the district.

### 3.5. Phylogenetic investigations

**Figure 9:**
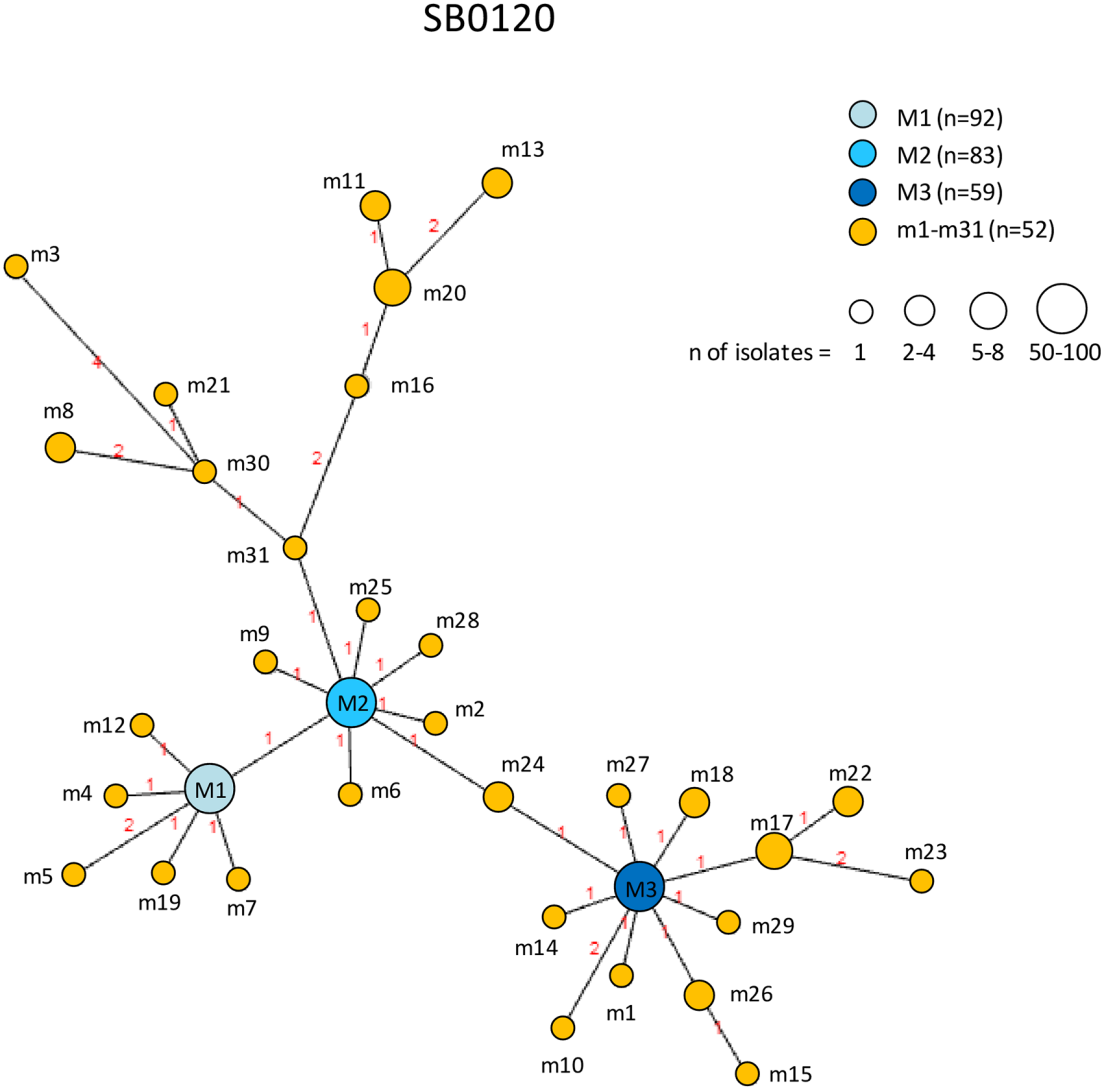
Minimum spanning tree of *Mycobacterium bovis* isolates belonging to spoligotype SB0120. The number of isolates (*n*) is indicated in the legend, in parentheses. Each circle represents a distinct 12-locus MIRU-VNTR code. The color of the circle represents a profile, as indicated in the legend: each of the major profiles has been assigned a shade of blue, whereas all minor profiles are shown in orange. The size of the circles is proportional to the number of isolates. Branch lengths correspond to the number of locus differences. Link labels refer to the distance matrix. The tree is based on PHYLOViZ Online.

**Figure 10:**
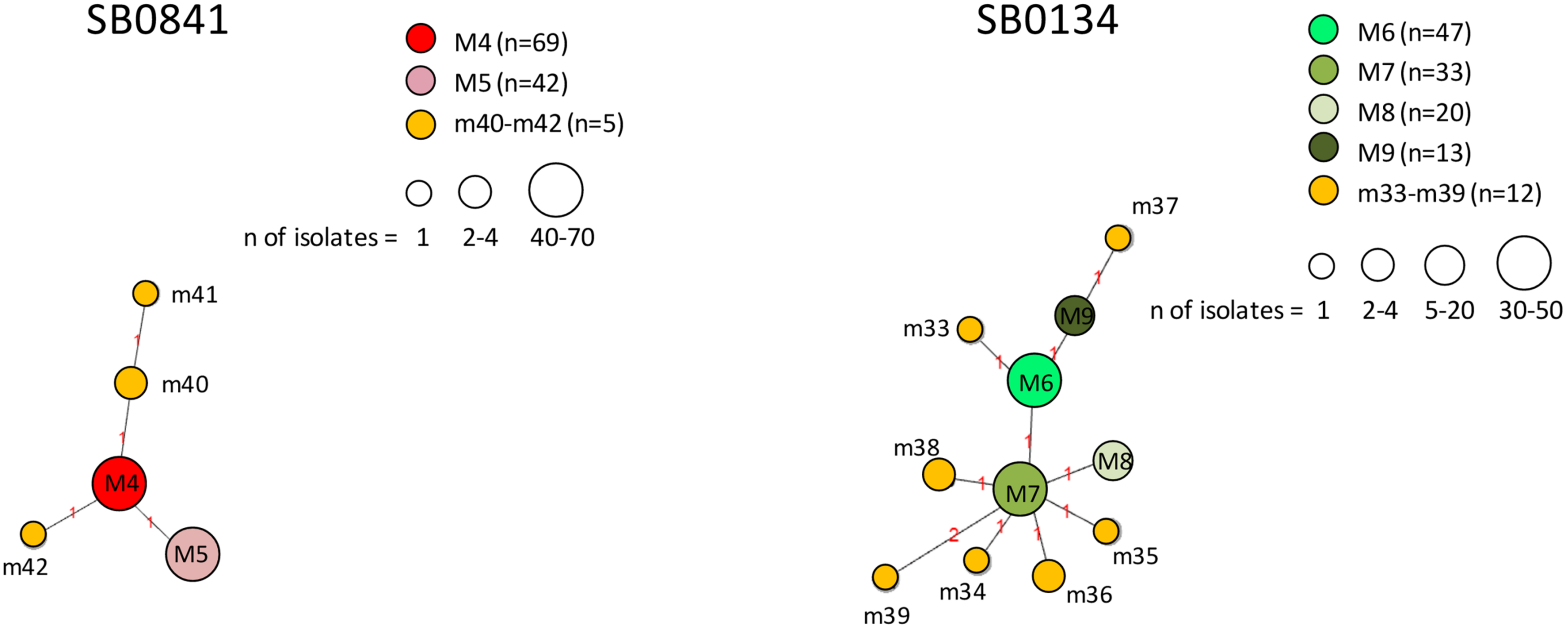
Minimum spanning trees of *Mycobacterium bovis* isolates belonging to spoligotypes SB0841 and SB0134. The number of isolates (*n*) is indicated in the legend, in parentheses. Each circle represents a distinct 12-locus MIRU-VNTR code. The color of the circle represents a profile, as indicated in the legend: each of the major profiles has been assigned a distinct color (red or a shade of green), whereas all minor profiles are shown in orange. The size of the circles is proportional to the number of isolates. Branch lengths correspond to the number of locus differences. Link labels refer to the distance matrix. The tree is based on PHYLOViZ Online.

Profiles obtained by combining the three predominant spoligotypes with their respective 12-locus MIRU-VNTR codes were used for the phylogenetic analysis. A total of 527 *M. bovis* isolates subtyped SB0120 (*n* = 286), SB0841 (*n* = 116), and SB0134 (*n* = 125) was analyzed. Results are shown in Figures 9, 10.

The genetic relationships between *M. bovis* subtype SB0120 isolates are shown in Figure 9. The minor profiles m1–m31 clustered around the three major profiles M1, M2, and M3. M2 was related to the largest number of minor profiles (14 profiles). In the SB0120 group, the profiles showing the highest number of differences between nodes are m8 and m20, which presented polymorphism at four loci, m11 at five loci, and m3 and m13 at six loci. All connected to major profile M2.

The genetic relationships between *M. bovis* profiles belonging to spoligotypes SB0841 and SB0134 are shown in Figure 10. Profiles m40, m41, and m42 branched off from major profile M4, profiles m34–m36, m38 and m39 from major profile M7, and profiles m33 and m37 from major profile M6. All minor profiles m33–m42 showed genetic differences at one or two loci.

## 4. Discussion

In previous studies, the district of Caronia—a small area of about 230 km^2^—emerged as having the highest number of TB cases among livestock in endemic Sicily (Amato et al., 2018; Marianelli et al., 2019). An intensive TB surveillance program implemented in Caronia between 2014 and 2018 offered us a rare opportunity to conduct an in-depth geo-epidemiological investigation of TB employing genotyping, geospatial (GIS), and phylogenetic analyses to investigate the transmission dynamics of *M. bovis* infection in the district and the role played by the ecosystem in the spread of the pathogen in livestock.

A large number of farms and animals were screened for TB infection. A total of 107 infected farms were found, and 589 *M. bovis* isolates were collected from livestock (cattle and black pigs).

Geospatial analysis of TB cases confirmed that the disease was spread widely across the district of Caronia. However, *M. bovis*-infected herds, many of which showed multiple and recurrent cases, were not randomly distributed but rather concentrated in specific areas or niches, according to the landscape structure. The highest number of TB cases mapped in a large hilly area in north-central Caronia, crossed by the Buzza stream. The geographic distribution of TB in cattle was similarly found not to be random in other TB-endemic regions, such as Zambia (Proud Tembo et al., 2020) and Mexico (Bastida et al., 2017), suggesting that both biotic and abiotic ecological factors play a role in the spatial distribution of TB in high-prevalence areas.

Combined genetic profiles have been widely used in epidemiological studies of *M. bovis* infection across the globe (Hauer et al., 2015; Carvalho et al., 2016; Egbe et al., 2017; Machado et al., 2018; Reis et al., 2020), and have proven useful in boosting the discriminatory power of *M. bovis* typing, thus providing more detailed data for the molecular-epidemiological analysis of this pathogen. In our 589 *M. bovis* isolates, we identified 62 different genetic profiles by combining 11 spoligotypes and 50 MIRU-VNTR codes.

Sicily has a long history of TB infection in cattle, especially in the province of Messina, according to the Epidemiological Veterinary Bulletins of Sicily.^1^ Our group has previously genotyped *M. bovis* isolates from livestock and wild animals throughout Sicily (Amato et al., 2018) and, more recently, from livestock in the province of Messina (Marianelli et al., 2019). We found a large number of genetically different *M. bovis* strains causing the disease. Numerous common genetic patterns were identified in farm animals from neighboring and even relatively distant districts of the province of Messina (Marianelli et al., 2019). A high number of different genetic profiles (10 major profiles and 52 minor profiles) were even found analyzing a single district of the province, namely the district of Caronia. It is probably that the high level of *M. bovis* heterogeneity we observed in both cattle and black pigs reared in Sicily is due to factors involving the geography of the territory, farming traditions and livestock trade that led to the failure of the national eradication program based on “test and cull” method in cattle and susceptible animals on the island.

Spoligotypes SB0120, SB0134, and SB0841 were the most prevalent in Caronia and affected both cattle and black pigs, in agreement with previous work by our group showing these spoligotypes to be the commonest in Sicily (Amato et al., 2018; Marianelli et al., 2019). The largest variety of spoligotypes was found in cattle. This is likely due both to the fact that cattle are the main host species for *M. bovis*, and to the fact that many more isolates from cattle were analyzed in this study. A larger number of black pigs should be studied to better understand the host susceptibility to *M. bovis* infection in our setting.

As expected, spatial analysis of the 10 most frequent genotypes (M1–M10) revealed that identical profiles were mostly found in livestock reared in the same or neighboring herds. Moreover, cattle and black pigs shared identical genetic profiles, confirming an intense intra- and inter-species *M. bovis* transmission in the area under study, in accordance with our previous study (Marianelli et al., 2019). Major profiles also showed geographic specificities in that they occupied specific spatial niches, as previously documented in Portugal (Reis et al., 2020), Northern Ireland (Hauer et al., 2015), Great Britan^2^ and France (Skuce et al., 2020).

Some genotypes (i.e., major profiles M3 and M5) were isolated mostly from farms situated west of the Caronia stream. It is likely that this mountainous area, characterized by steep slopes, has created physical barriers that prevented these profiles from spreading east. The sporadic TB cases due to M3 and M5 recorded in the eastern part of the district may be attributable to local livestock trading. Other profiles (i.e., major profiles M1, M2, M4, M7, and M10) were isolated from numerous herds grouped in a large hilly area ranging from the north to the center of the district, along the Buzza stream. The high density of herds both along the Buzza and in the surrounding hills may have promoted contact between infected and uninfected animals, especially in the dry season, at watering points along the stream, and may thus explain the high prevalence of *M. bovis* infection recorded in that area and the co-circulation of a high number of different major genotypes. Interestingly, profiles M6 and M8 were both found only in the eastern part of the district, east of a long rocky ridge, and grouped mostly on a hilly plateau where livestock share pastures. The physical barrier of the long rocky ridge may have prevented indirectly the spread of these two profiles to western Caronia, by restricting the movement of infected animals—both livestock and wildlife. The lack of the livestock trade between the eastern and western part of Caronia may also explain the above results.

The geographical distribution of major *M. bovis* genotypes observed in the Caronia district strongly suggests that geography and landscape morphology play a key role in the spread of this pathogen. Mountains with steep slopes and rocky ridges constitute physical barriers to animal moving and grazing, and consequently to the spread of infection. Conversely, pastures, plateaus and streams favor animal aggregation and thus increase the risk of inter- and intra-species contagion. A recent study conducted in Zambia identified the sharing of grazing grounds and drinking water as the most important risk factors in the spread of TB (Proud Tembo et al., 2020). The importance of water resources as indirect routes of cross-species TB transmission was highlighted also by others (Barasona et al., 2017).

Farming methods can significantly contribute to increase the risk of inter- and intra-herd transmission of TB. In Sicily, livestock is raised in small-scale farms, where multiple animal species are often reared together. Cattle and black pigs are the most common species of livestock in the area. Extensive agricultural practices are used for both cattle and black pigs throughout the year. Cattle are reared in grasslands and natural pastures, whereas black pigs are raised in free or semi-free roaming conditions in the woods and natural pastures of the Nebrodi Park. Cattle and black pigs may therefore come into contact with each other at shared pastures, waterholes and feeding sites, where infected animals may transmit the infection to healthy animals, be it within or between farms and/or species.

Major profiles, such as M1 and M3–M5, were isolated from non-neighboring herds as well, some of which quite distant from one another. These data point to the presence of multiple sources of infection. Presumably, beyond the above-mentioned extensive and multi-species livestock rearing systems, local livestock trade, livestock auctions and the exchange of stud animals between farms, which reflect local culture, may explain cross-contamination across the district. These are all factors known to have significant impacts on *M. bovis* transmission (Green et al., 2008; Martínez-López et al., 2014; Palisson et al., 2016; Fielding et al., 2020).

Contact with wildlife is regarded an additional factor driving the spread of TB infection (Proud Tembo et al., 2020; Reis et al., 2021). The district of Caronia is part of the Nebrodi Park. Wild mammals living in the park are likely to play an important role in the epidemiology of *M. bovis* infection. Caronia is mostly mountainous and covered in thick vegetation which provides habitat for hedgehogs, wildcats, martens and dormice. Unfortunately, the literature offers little information on the potential role of these wild mammals in *M. bovis* transmission to livestock. Delahay et al. (2002) reviewed the role of several wild mammals—i.e. badgers, foxes, stoats, weasels, otters, deer, lagomorphs and other small mammals—in the transmission of *M. bovis* to cattle in the United Kingdom. Although they documented *M. bovis* infection in several mustelids (i.e., stoats and weasels), they found no wild mammal, apart from the badger, to represent a significant, self-maintaining reservoir of TB infection (Delahay et al., 2002). In New Zealand, Lugton et al. (1995) isolated this pathogen from European hedgehogs with lung lesions, concluding that these animals might constitute a reservoir for the disease. In France, Michelet et al. (2018) described *M. bovis* infection in red foxes. Ultimately, however, the lack of sufficient data and proper surveillance systems on many wild mammals hampers the understanding of their epidemiological role in TB infection, and further studies are therefore needed. For the time being, not having considered wildlife in the present study, we are unable to draw any conclusions on the possible involvement of wildlife in the transmission of TB infection in the district of Caronia. We are planning a study to address this matter.

Contrary to the 10 major profiles, most of the remaining 52 minor profiles affected only one or two animals each. The minor profiles were genetically related to the major profiles, mainly to M2, and in most cases presented genetic differences at only one or two loci. It has been suggested that the presence of single or double locus variations is consistent with a clonal expansion of a founder strain (Reis et al., 2020). It is therefore possible that the minor profiles belonging to spoligotypes SB0120, SB0841, SB0134 are derived from M2, M4 and M6/M7, respectively. Yet, since multi-locus VNTR analysis and similar typing methods are often confounded by homoplasy (evolutionary reversals, convergences, parallelisms and lateral gene transfer), which complicates the ability to trace patterns of descent (Pearson et al., 2009), the precision and accuracy of the above results may be affected. Homoplasy can be mitigated by the use of a large number of loci (e.g., 24) (Reyes et al., 2012). In addition, integration of sub-genomic genotyping methods with whole genome sequencing (WGS) will enable highly accurate phylogeny estimations (Pearson et al., 2009). MIRU-VNTR analysis can therefore be used as first-order analysis to characterize *M. bovis* from a specific area. WGS can subsequently be used to study the transmission dynamics of observed epidemics and to quantify the extent and direction of transmission at landscape level (Biek et al., 2012). This landscape genetics approach will enable researchers to assess how environmental variables contribute to the distribution of *M. bovis* infection and influence microevolutionary processes of *M. bovis*, as previously discussed by Biek and Real (2010).

As far as the geographical distribution of minor profiles is concerned, the only minor profiles to form groups—m49 and m50—affected 11 and 10 animals, respectively (as the others numbered a couple of cases each). These profiles were localized mainly in the central and eastern parts of Caronia. Conceivably, the analysis of a larger number of TB cases caused by minor profiles would reveal the formation of groups and link them to the landscape, as observed for the major profiles. Future investigations and regular updates will provide a better understanding of their clustering behavior.

The low number of cases due to minor profiles, coupled with their scattered geographical distribution, may suggest that the minor profiles originated more recently than the more firmly established major profiles, or indicate a reduced transmissibility compared to the major profiles.

In conclusion, this study paints a complex picture of the epidemiological dynamics of TB in this very limited geographical area. Numerous *M. bovis* genotypes, transmitted within and between neighboring and non-neighboring herds have been identified, and several risk factors providing insights into TB transmission and persistence have been described. The landscape structures—i.e. slopes, rocky ridges, hills, steams, plateaus, shared watering points and pastures, local livestock trading, extensive farming practices in multispecies herds, may all have contributed to the spread and maintenance of *M. bovis* infection in Caronia. The large number of different *M. bovis* genotypes here described makes the epidemiological situation of TB in Caronia unique. Geospatial analysis coupled with genetic analysis has proven useful in understanding the variations in the occurrence of TB and the distribution of *M. bovis* genotypes across Caronia, as well as in identifying vulnerable animal populations and at-risk geographic niches in the district. Our results can help in the planning of effective, tailor-made TB surveillance plans in the at-risk subareas of the district, especially those crossed by streams and characterized by a high density of farms. A study of TB among the wildlife of the Nebrodi Park will be conducted as a next step for a more comprehensive understanding of the transmission dynamics of *M. bovis* in livestock in this setting.

## Data availability statement

The original contributions presented in the study are included in the article/Supplementary material, further inquiries can be directed to the corresponding author.

## Ethics statement

Ethical review and approval was not required for the animal study because sample were collected during routine abattoir inspections within official contexts based on the national legislation for cattle and research projects funded by the Italian Ministry of health for the Sicilian black pigs. No animals were slaughtered for the purposes of this study and no permission was needed.

## Author contributions

CM conceived and designed the study and wrote the paper. FP, DI, MP, and VM collected the data. CM and VV performed the analysis and interpretation of data. All authors contributed to the article and approved the submitted version.

## Conflict of interest

The authors declare that the research was conducted in the absence of any commercial or financial relationships that could be construed as a potential conflict of interest.

## Publisher’s note

All claims expressed in this article are solely those of the authors and do not necessarily represent those of their affiliated organizations, or those of the publisher, the editors and the reviewers. Any product that may be evaluated in this article, or claim that may be made by its manufacturer, is not guaranteed or endorsed by the publisher.

## Footnotes

^1^ https://sites.google.com/a/izssicilia.it/izs/siti-tematici/bollettino-epidemiologico-veterinario-della-sicilia

^2^ https://www.gov.uk/government/publications/bovine-tb-epidemiology-and-surveillance-in-great-britain-2019
