# Supplementary material for: Geo-epidemiology of animal tuberculosis and Mycobacterium bovis genotypes in livestock in a small, high-incidence area in Sicily, Italy
Source: Front Microbiol. 2023 Mar 17;14:1107396. doi: 10.3389/fmicb.2023.1107396 (PMC10063800; doi:10.3389/fmicb.2023.1107396)
Supplement: Supplementary file 1 [file Table_1.docx]

**Table S1. Minor *M. bovis* genetic profiles by host, number of farms and year of isolation**

| **Spoligotype** | **12-locus MIRU-VNTR code** | **Profile*** | **Host (n)** | **Number of farms** | **Year of isolation** |
| --- | --- | --- | --- | --- | --- |
| SB0120 | 4, 5, 5, 3, 3, 10, 4, 4, 4, 3, 6, 4 | m1 | cow (1) | 1 | 2016 |
|  | 3, 3, 5, 3, 3, 10, 4, 4, 4, 3, 6, 4 | m2 | cow (1) | 1 | 2016 |
|  | 6, 4, 5, 3, 3, 10, 2, 5, 4, 3, 6, 2 | m3 | cow (1) | 1 | 2016 |
|  | 3, 3, 5, 3, 3, 10, 6, 4, 4, 3, 6, 3 | m4 | cow (1) | 1 | 2016 |
|  | 3, 3, 5, 3, 3, 10, 6, 4, 4, 3, 9, 4 | m5 | cow (1) | 1 | 2017 |
|  | 3, 3, 5, 3, 3, 10, 4, 4, 4, 3, 4, 5 | m6 | cow (1) | 1 | 2016 |
|  | 3, 3, 5, 3, 3, 10, 6, 4, 4, 3, 7, 5 | m7 | cow (1) | 1 | 2015 |
|  | 5, 4, 5, 3, 3, 10, 4, 4, 4, 1, 7, 5 | m8 | cow (2) | 2 | 2015, 2016 |
|  | 3, 3, 5, 3, 3, 10, 4, 4, 4, 3, 7, 5 | m9 | cow (1) | 1 | 2016 |
|  | 5, 5, 5, 3, 3, 10, 4, 4, 4, 3, 5, 5 | m10 | cow (1) | 1 | 2015 |
|  | 5, 5, 5, 3, 4, 10, 4, 5, 4, 2, 6, 5 | m11 | cow (2)  black pig (1) | 2 | 2016, 2017 |
|  | 3, 3, 5, 3, 3, 10, 6, 4, 4, 2, 6, 5 | m12 | cow (1) | 1 | 2016 |
|  | 5, 4, 5, 3, 4, 10, 4, 5, 2, 3, 6, 5 | m13 | cow (2) | 2 | 2014, 2016 |
|  | 4, 5, 5, 3, 3, 10, 4, 4, 2, 3, 6, 5 | m14 | cow (1) | 1 | 2016 |
|  | 4, 5, 5, 3, 3, 10, 3, 4, 2, 3, 6, 5 | m15 | cow (1) | 1 | 2016 |
|  | 5, 3, 5, 3, 4, 10, 4, 5, 4, 3, 6, 5 | m16 | cow (1) | 1 | 2015 |
|  | 4, 5, 5, 3, 3, 10, 4, 3, 4, 3, 6, 5 | m17 | cow (5)  black pig (1) | 2 | 2016, 2017 |
|  | 4, 5, 5, 3, 3, 10, 4, 2, 4, 3, 6, 5 | m18 | cow (3) | 1 | 2016 |
|  | 3, 3, 5, 3, 3, 10, 6, 2, 4, 3, 6, 5 | m19 | cow (1) | 1 | 2016 |
|  | 5, 5, 5, 3, 4, 10, 4, 5, 4, 3, 6, 5 | m20 | cow (8) | 3 | 2015, 2016, 2018 |
|  | 5, 4, 5, 3, 3, 10, 4, 3, 4, 3, 6, 5 | m21 | cow (1) | 1 | 2015 |
|  | 4, 4, 5, 3, 3, 10, 4, 3, 4, 3, 6, 5 | m22 | cow (2) | 1 | 2016 |
|  | 5, 5, 5, 3, 3, 8, 4, 3, 4, 3, 6, 5 | m23 | cow (1) | 1 | 2016 |
|  | 4, 3, 5, 3, 3, 10, 4, 4, 4, 3, 6, 5 | m24 | cow (2) | 1 | 2015, 2016 |
|  | 3, 3, 5, 3, 3, 10, 3, 4, 4, 3, 6, 5 | m25 | cow (1) | 1 | 2015 |
|  | 4, 5, 5, 3, 3, 10, 3, 4, 4, 3, 6, 5 | m26 | cow (2) | 2 | 2015, 2016 |
|  | 4, 5, 5, 3, 3, 10, 5, 4, 4, 3, 6, 5 | m27 | cow (1) | 1 | 2016 |
|  | 3, 3, 5, 3, 3, 7, 4, 4, 4, 3, 6, 5 | m28 | cow (1) | 1 | 2015 |
|  | 4, 5, 5, 3, 3, 5, 4, 4, 4, 3, 6, 5 | m29 | cow (1) | 1 | 2017 |
|  | 5, 4, 5, 3, 3, 10, 4, 4, 4, 3, 6, 5 | m30 | cow (1) | 1 | 2015 |
|  | 5, 3, 5, 3, 3, 10, 4, 4, 4, 3, 6, 5 | m31 | cow (1) | 1 | 2016 |
| SB0133 | 5, 5, 5, 3, 3, 10, 4, 4, 4, 3, 6, 5 | m32 | cow (5) | 2 | 2016 |
| SB0134 | 5, 2, 5, 3, 4, 10, 3, 5, 4, 3, 6, 5 | m33 | cow (1) | 1 | 2015 |
|  | 5, 4, 5, 3, 4, 10, 4, 5, 4, 3, 6, 7 | m34 | cow (1) | 1 | 2015 |
|  | 5, 3, 5, 3, 4, 10, 4, 5, 4, 3, 6, 5 | m35 | cow (1) | 1 | 2015 |
|  | 5, 4, 5, 3, 4, 10, 4, 5, 4, 3, 8, 5 | m36 | cow (4) | 1 | 2016, 2017 |
|  | 5, 4, 6, 3, 4, 10, 3, 5, 4, 3, 5, 5 | m37 | cow (1) | 1 | 2016 |
|  | 5, 4, 5, 3, 4, 10, 5, 5, 4, 3, 6, 5 | m38 | black pig (3) | 1 | 2018 |
|  | 4, 4, 5, 3, 4, 10, 4, 5, 4, 3, 6, 5 | m39 | cow (1) | 1 | 2018 |
| SB0841 | 4, 5, 5, 3, 3, 10, 4, 4, 4, 3, 6, 5 | m40 | cow (3) | 3 | 2015, 2016 |
|  | 4, 5, 5, 3, 3, 10, 4, 4, 2, 3, 6, 5 | m41 | cow (1) | 1 | 2015 |
|  | 5, 5, 5, 3, 3, 10, 4, 1, 4, 3, 6, 5 | m42 | cow (1) | 1 | 2016 |
| SB0850 | 4, 5, 5, 3, 3, 10, 4, 4, 3, 3, 5, 5 | m43 | cow (1) | 1 | 2015 |
| SB1167 | 3, 3, 5, 3, 3, 10, 6, 4, 4, 3, 6, 5 | m44 | cow (1) | 1 | 2016 |
| SB1305 | 6, 4, 3, 3, 3, 10, 1, 5, 4, 3, 7, 2 | m45 | cow (1) | 1 | 2016 |
| SB1564 | 3, 3, 5, 3, 3, 10, 4, 4, 4, 3, 7, 5 | m46 | cow (1) | 1 | 2014 |
|  | 3, 3, 5, 3, 3, 10, 3, 4, 4, 3, 6, 5 | m47 | cow (1) | 1 | 2015 |
|  | 2, 3, 5, 3, 3, 10, 4, 4, 4, 3, 6, 5 | m48 | cow (2) | 1 | 2016 |
|  | 3, 3, 5, 3, 3, 10, 4, 4, 4, 3, 6, 5 | m49 | cow (11) | 8 | 2015, 2016 |
| SB1566 | 3, 3, 5, 3, 3, 10, 4, 4, 4, 3, 6, 5 | m50 | cow (10) | 4 | 2016 |
| SB2368 | 5, 5, 5, 3, 3, 10, 4, 4, 4, 3, 5, 5 | m51 | cow (1) | 1 | 2015 |
| SB2473 | 6, 4, 3, 3, 3, 10, 2, 5, 4, 3, 7, 2 | m52 | cow (5) | 1 | 2015 |

*Profiles were obtained by combining spoligotypes and 12-locus MIRU-VNTR codes (see Materials and Methods); n = number of isolates. The minor genetic profiles are the less common ones.
